# Comparison of non-contrast CT, CT perfusion, and CT angiography for predicting delayed cerebral ischemia after aneurysmal subarachnoid hemorrhage

**DOI:** 10.1186/s13244-026-02238-z

**Published:** 2026-03-03

**Authors:** Xin Yao, Haifeng Cheng, Chen Yang, Xiaojun Hao, Xintong Zhao, Xinggen Fang, Yunfeng Zhou, Chao Zhang

**Affiliations:** 1 Department of Radiology, Yijishan Hospital of Wannan Medical College, Wuhu, People’s Republic of China; 2 Department of Neurosurgery, Yijishan Hospital of Wannan Medical College, Wuhu, People’s Republic of China

**Keywords:** Aneurysmal subarachnoid hemorrhage, Delayed cerebral ischemia, Non-contrast CT, CT perfusion, CT angiography

## Abstract

**Objective:**

Non-contrast CT (NCCT), CT perfusion (CTP), and CT angiography (CTA) are recommended for predicting delayed cerebral ischemia (DCI) after aneurysmal subarachnoid hemorrhage (aSAH). However, not all patients can undergo all three examinations on admission. We aimed to compare the predictive abilities of NCCT, CTP, and CTA for DCI.

**Materials and methods:**

This retrospective study enrolled consecutive aSAH patients admitted to our center between November 2015 and September 2023. NCCT, CTP, and CTA models were constructed using logistic regression analyses adjusted for confounders. The model performances were assessed by discrimination and calibration. Internal validation was conducted using bootstrapping. The predictive abilities were further evaluated in subgroup analyses.

**Results:**

A total of 950 patients (median [IQR] age: 59 [51–68] years; 651 women) were enrolled, of whom 246 (25.9%) developed DCI. The NCCT model had an area under the curve (AUC) of 0.837 (95% CI: 0.808–0.866), and was superior to the CTP (AUC: 0.783; 95% CI: 0.748–0.818; *p* < 0.001) and CTA (AUC: 0.760; 95% CI: 0.723–0.797; *p* < 0.001) models. All three models had good calibration ability (all *p* > 0.05). Internal validation showed satisfactory discrimination ability (optimism-adjusted AUC: 0.840 for the NCCT model, 0.785 for the CTP model, and 0.761 for the CTA model). The NCCT and CTP models exhibited similar predictive abilities (AUC: 0.763 vs. 0.735; *p* = 0.399) in the poor-grade aSAH (World Federation of Neurological Surgeons 4–5) group.

**Conclusion:**

The NCCT model performed better than the CTP and CTA models for predicting DCI and was comparable to the CTP model in poor-grade aSAH patients.

**Critical relevance statement:**

For most aneurysmal subarachnoid hemorrhage patients, non-contrast CT performed at emergency admission is sufficient to evaluate disease severity and reliably predict the risk of delayed cerebral ischemia.

**Key Points:**

- More straightforward and reliable indicators are required to facilitate early delayed cerebral ischemia prediction.
- The non-contrast CT model, utilizing admission variables, was most predictive of delayed cerebral ischemia.
- Non-contrast CT at admission reliably predicts delayed cerebral ischemia risk and severity in most aneurysmal subarachnoid hemorrhage patients.

**Graphical Abstract:**

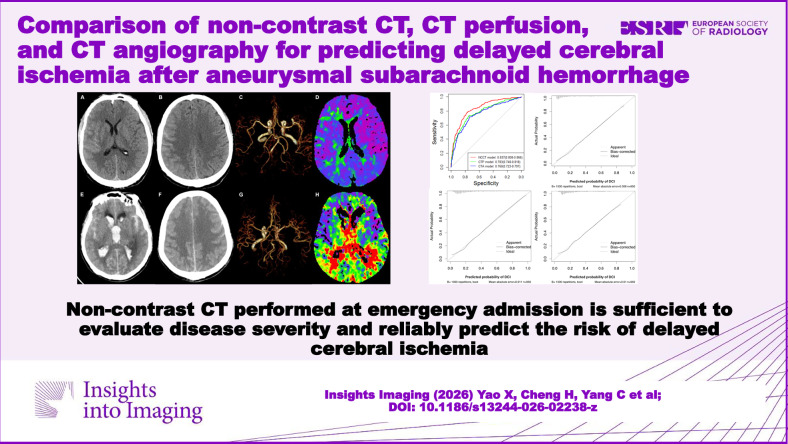

## Introduction

Delayed cerebral ischemia (DCI) occurs in 20%–30% of aneurysmal subarachnoid hemorrhage (aSAH) patients [1] and is a major, potentially modifiable contributor to late disability and mortality [2, 3]. Timely and precise prediction of DCI is the first step in guiding personalized treatment [1, 3]. In clinical practice, the assessment of aSAH severity is initially based on neurological examinations [4]. However, the proportion of poor-grade aSAH survivors has increased in recent years [5], and the clinical changes in these patients may be difficult to detect and have limited prognostic value [1, 3]. Therefore, more effective predictive indicators are needed to identify high-risk patients for developing DCI.

Through studies on the multifactorial mechanism of DCI, several risk factors based on multimodal imaging modalities have been identified. The initial amount of hemorrhage [6–8], the presence of acute hydrocephalus [9], and the severity of early brain edema [10] can be qualitatively and semi-quantitatively assessed using non-contrast CT (NCCT). Aneurysm morphology and cerebral vasospasm (CVS) can be determined using CT angiography (CTA) [11, 12]. These modalities are quick to perform and provide images that are easy to interpret. In recent years, research on CT perfusion (CTP) has rapidly expanded. CTP can detect early cerebral microcirculatory dysfunction after aSAH and may serve as a predictive tool for DCI development [13].

For patients with ischemic stroke, several studies have indicated that NCCT, CTP, and CTA exhibit comparable predictive values [14, 15]. In patients with aSAH, the diagnostic value of CTP for DCI is superior to that of NCCT and CTA during clinical deterioration [16]. However, though the guideline recommends the use of NCCT, CTP, and CTA for the prediction of DCI [3], not all patients are able to undergo all three imaging modalities upon emergency admission. Moreover, it remains unclear whether the predictive performances of the three modalities are comparable. In the present study, we constructed NCCT, CTA, and CTP models and compared the abilities of the three models to predict DCI in aSAH patients.

## Materials and methods

### Study population

**Fig. 1:**
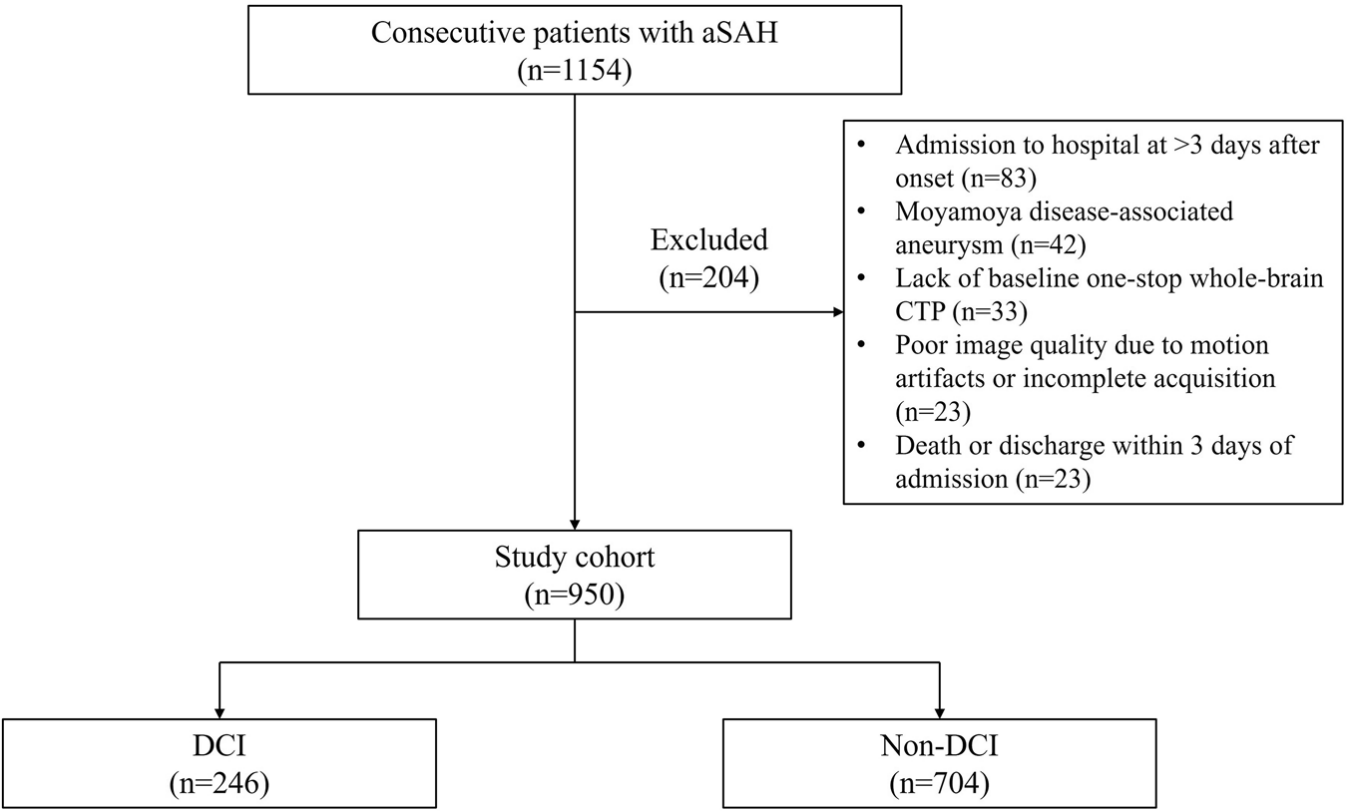
Flowchart of patient selection. aSAH indicates aneurysmal subarachnoid hemorrhage; CTP, CT perfusion; and DCI, delayed cerebral ischemia

This retrospective observational study was approved by the institutional review board of our institution, and the requirement for patient consent was waived. Using our prospective database for aSAH, we retrospectively reviewed all consecutive patients who had confirmed aSAH on NCCT and digital subtraction angiography within 3 days after onset and were admitted to our single stroke center between November 2015 and September 2023. The inclusion criteria were as follows: (a) age > 18 years; and (b) aneurysm treated with coiling or clipping. The exclusion criteria were as follows: (a) admission to hospital at > 3 days after onset; (b) moyamoya disease-associated aneurysm; (c) lack of baseline one-stop whole-brain CTP (including NCCT, CTP, and CTA); (d) poor image quality due to motion artifacts or incomplete acquisition; and (e) death or discharge within 3 days of admission. A flowchart of the patient selection is shown in Fig. 1.

### Data collection

Baseline hospitalization data were collected from the aSAH database. The demographic characteristics included age, sex, and hypertension. The clinical severity of aSAH on admission was evaluated using the World Federation of Neurological Surgeons (WFNS) grading scale and dichotomized into good-grade (WFNS 1–3) or poor-grade (WFNS 4–5) aSAH, as previously reported [4].

**Fig. 2:**
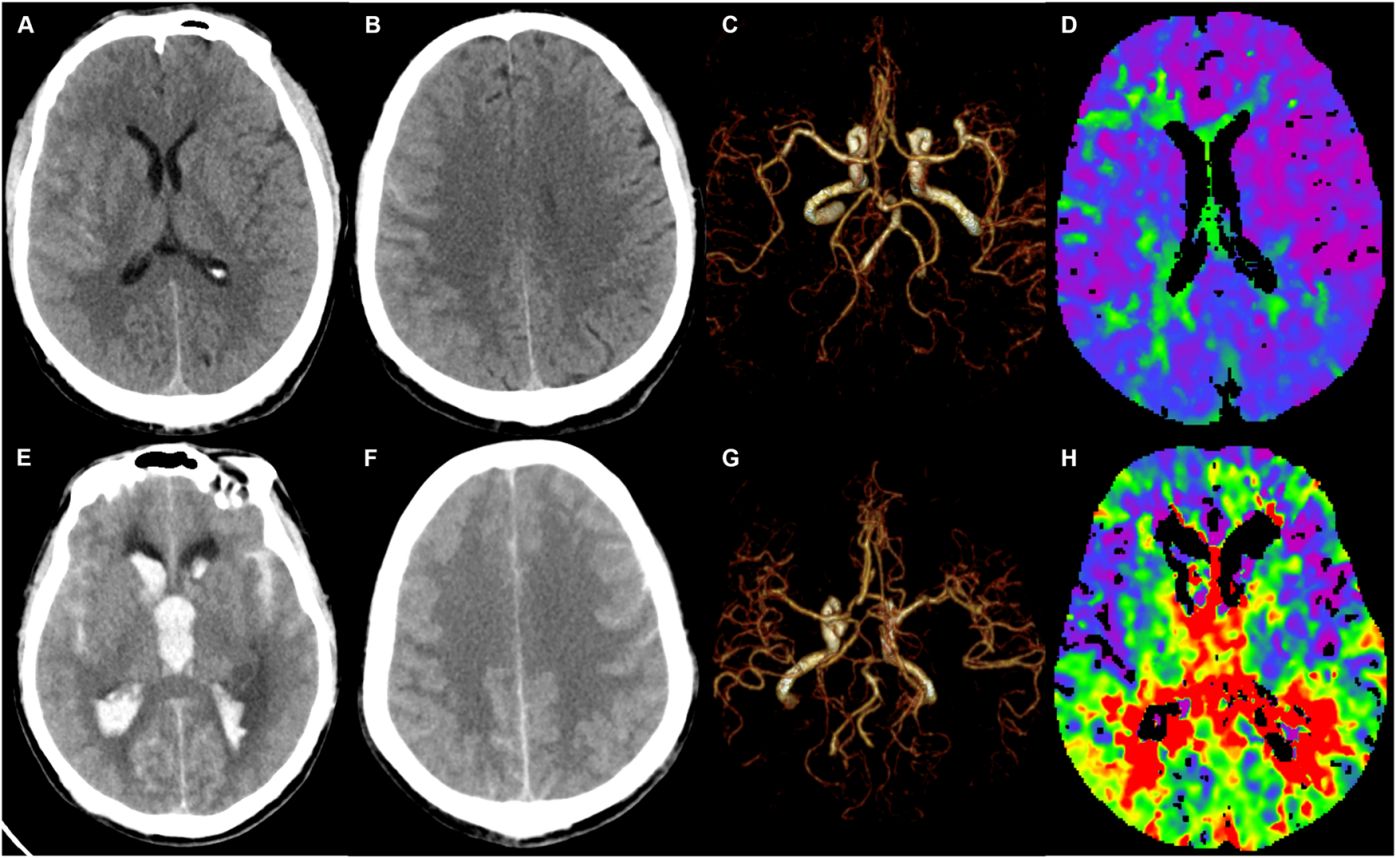
One-stop whole-brain CT perfusion images of patients with and without delayed cerebral ischemia (DCI). A 53-year-old male with evidence of DCI. **A**,** B** Non-contrast computed tomography (NCCT) demonstrated a modified Fisher Score (mFS) of 1 and a Subarachnoid Hemorrhage Early Brain Edema Score (SEBES) of 1, with no signs of intraventricular hemorrhage (IVH), intracranial hematoma, or acute hydrocephalus. **C** Computed tomography angiography (CTA) confirmed the presence of a right middle cerebral artery aneurysm. **D** TMax perfusion imaging revealed comparatively lower perfusion in the right cerebral hemisphere relative to the left. A 62-year-old male patient presented with DCI. **E**,** F** NCCT revealed an mFS of 4 and an SEBES of 4, along with the presence of IVH and acute hydrocephalus. **G** CTA identified a left posterior communicating artery aneurysm. **H** TMax perfusion imaging further demonstrated decreased perfusion across both cerebral hemispheres diffusely

Based on recent research regarding imaging predictors for DCI, the following imaging data were collected: modified Fisher Score (mFS) [6], intraventricular hemorrhage (IVH) [7], intracranial hematoma [8], acute hydrocephalus [9], and Subarachnoid Hemorrhage Early Brain Edema Score (SEBES) [10] were evaluated on NCCT; ruptured aneurysm size and location [11] and CVS [12] were determined on CTA; and cerebral blood flow (CBF), cerebral blood volume (CBV), mean transit time (MTT), time to delay (TTD), time to start (TTS), and transit time to the center of the impulse response function (TMax) were obtained on CTP. The one-stop whole-brain CTP protocol and CTP post-processing method were described previously [17]. The NCCT, CTA, and CTP data were separately assessed by three neuroradiologists who were blinded to the results of the other two imaging examinations and clinical information. Any differences in their assessments were resolved through consensus. Two representative cases are shown in Fig. 2.

### Definition of DCI

The presence of DCI was assessed using the 2010 Multidisciplinary Research Group definition [18], which encompasses (a) onset of focal neurological impairment or decrease of ≥ 2 points on the Glasgow Coma Scale, lasting for at least 1 hour, not immediately caused by an apparent post-aneurysm occlusion and not attributed to other conditions; and (b) presence of a cerebral infarction on CT or MRI within 6 weeks post-onset that was absent on imaging examinations between 24 and 48 hours post-aneurysm occlusion and was not linked to endovascular treatment. Hypodensities on CT resulting from a ventricular catheter or an intraparenchymal hematoma were not considered cerebral infarctions from DCI.

### Statistical analysis

Statistical analyses were conducted using SPSS version 25.0 (IBM Corporation) and R version 3.4.2 (R Foundation for Statistical Computing). Significance was defined as a two-tailed value of *p* < 0.05. Since all continuous variables had non-normal distributions, they were described as the median and interquartile range, and were compared using the Mann–Whitney *U*-test. All categorical variables were expressed as frequency and percentage, and were compared using the Pearson chi-square test. Imaging variables with values of *p* < 0.1 in the univariate analyses were entered into the logistic regression analysis with a forward stepwise selection. The NCCT, CTA, and CTP models were then constructed with adjustment for known prognostic variables (age, hypertension, WFNS, and treatment modality) based on the SAHIT multinational cohort study [19]. The predictive abilities of the three models were assessed by discrimination and calibration. Model discrimination was evaluated by receiver operating characteristic (ROC) curve and area under the curve (AUC) analysis. An AUC of 0.70–0.79 was considered good discrimination, and an AUC of 0.80–0.89 was considered excellent discrimination. The Delong test was used to compare the AUC values. Model calibration was evaluated by the Hosmer–Lemeshow test and visualized by calibration plots, with a value of *p* > 0.05 considered to indicate good calibration. Internal validation was conducted using the bootstrap method to correct for overestimation and obtain the optimism-adjusted AUC [20]. The predictive abilities were further evaluated by subgroup analyses in good-grade and poor-grade aSAH patients.

## Results

### Patient characteristics

Of the 1154 consecutive patients in the aSAH database, 950 were finally included in the analysis. The median age was 59 years (IQR: 51–68 years), and 651 (68.5%) were women. On admission, 697 patients (73.4%) were identified as good-grade aSAH, and 253 patients (26.6%) were identified as poor-grade aSAH. Regarding treatment, 905 patients (95.3%) underwent coil embolization, and 45 patients (4.7%) underwent microsurgical clipping. DCI developed in 246 patients (25.9%). The patients with DCI were older (65 years [56–73 years] vs. 56 years [51–66 years]; *p* < 0.001) and more likely to have hypertension (54.5% vs. 41.3%; *p* < 0.001) and higher WFNS (54.1% vs. 17.0%; *p* < 0.001) than the patients without DCI (Table 1).

**Table 1:** Univariate analysis of variables between the non-DCI and DCI groups

Characteristic	Non-DCI (*n* = 704)	DCI (*n* = 246)	*p*
Demographic and clinical data			
Age, years, median (IQR)	56 (51, 66)	65 (56, 73)	< 0.001
Sex, woman, *n* (%)	471 (66.9)	180 (73.2)	0.068
Hypertension, *n* (%)	291 (41.3)	134 (54.5)	< 0.001
WFNS, *n* (%)			< 0.001
Poor-grade (4–5)	120 (17.0)	133 (54.1)	
Good-grade (1–3)	584 (83.0)	113 (45.9)	
NCCT data			
mFS, *n* (%)			< 0.001
High (3–4)	321 (45.6)	198 (80.5)	
Low (0–2)	383 (54.4)	48 (19.5)	
SEBES, *n* (%)			
High (3–4)	68 (9.7)	68 (27.6)	< 0.001
Low (0–2)	636 (90.3)	178 (72.4)	
Intracranial hematoma, *n* (%)	85 (12.1)	101 (41.1)	< 0.001
IVH, *n* (%)	473 (67.2)	222 (90.2)	< 0.001
Acute hydrocephalus, *n* (%)	194 (27.6)	174 (70.7)	< 0.001
CTP data			
CBF, ml/100 g/min, median (IQR)	65.63 (60.38, 71.34)	60.84 (55.11, 67.04)	0.003
CBV, ml/100 g, median (IQR)	4.00 (3.77, 4.32)	3.88 (3.66, 4.24)	< 0.001
MTT, s, median (IQR)	4.19 (3.84, 4.68)	4.52 (4.07, 5.21)	< 0.001
TTS, s, median (IQR)	0.43 (0.26, 0.67)	0.73 (0.44, 1.05)	< 0.001
TTD, s, median (IQR)	4.03 (3.51, 4.63)	4.82 (4.07, 5.62)	< 0.001
TMax, s, median (IQR)	1.96 (1.57, 2.36)	2.48 (1.97, 3.11)	< 0.001
CTA data			
Anterior circulation, *n* (%)	642 (91.2)	220 (89.4)	0.412
Aneurysm size, mm, median (IQR)	4.5 (3.5, 6.2)	5.1 (3.9, 7.2)	< 0.001
CVS, *n* (%)	573 (81.4%)	197 (80.1%)	0.652
Treatment method			0.027
Coiling, *n* (%)	677 (96.2%)	228 (92.7%)	
Clipping, *n* (%)	27 (3.8%)	18 (7.3%)	

*CBF* cerebral blood flow, *CBV* cerebral blood volume, *CTA* CT angiography, *CTP* CT perfusion, *CVS* cerebral vasospasm, *DCI* delayed cerebral ischemia, *IVH* intraventricular hemorrhage, *mFS* modified Fisher Score, *MTT* mean transit time, *NCCT* non-contrast CT, *SEBES* Subarachnoid Hemorrhage Early Brain Edema Score, *TMax* transit time to the center of the impulse response function, *TTD* time to delay, *WFNS* World Federation of Neurosurgery Scale

### Univariate analyses of imaging variables associated with DCI

DCI was more frequent in patients with IVH (90.2% vs. 67.2%; *p* < 0.001), intracranial hematoma (41.1% vs. 12.1%; *p* < 0.001), and acute hydrocephalus (70.7% vs. 27.6%; *p* < 0.001), and in patients with larger aneurysm size (5.1 mm [3.9–7.2 mm] vs. 4.5 mm [3.5–6.2 mm]; *p* < 0.001), higher mFS (80.5% vs. 45.6%; *p* < 0.001), higher SEBES (80.5% vs. 45.6%; *p* < 0.001), longer MTT (4.52 s [4.07–5.21 s] vs. 4.19 s [3.84–4.68 s]; *p* < 0.001), longer TTS (0.73 s [0.44–1.05 s] vs. 0.43 s [0.26–0.67 s]; *p* < 0.001), longer TTD (4.82 s [4.07–5.62 s] vs. 4.03 s [3.51–4.63 s]; *p* < 0.001), longer TMax (2.48 s [1.97–3.11 s] vs. 1.96 s [1.57–2.36 s]; *p* < 0.001), lower CBF (60.84 mL/100 g/min [55.11–67.04 mL/100 g/min] vs. 65.63 mL/100 g/min [60.38–71.34 mL/100 g/min]; *p* = 0.003), and lower CBV (3.88 mL/100 g [3.66–4.24 mL/100 g] vs. 65.63 mL/100 g [60.38–71.34 mL/100 g]; *p* = 0.001) (Table 1).

### Predictive abilities of the NCCT, CTA, and CTP models

**Fig. 3:**
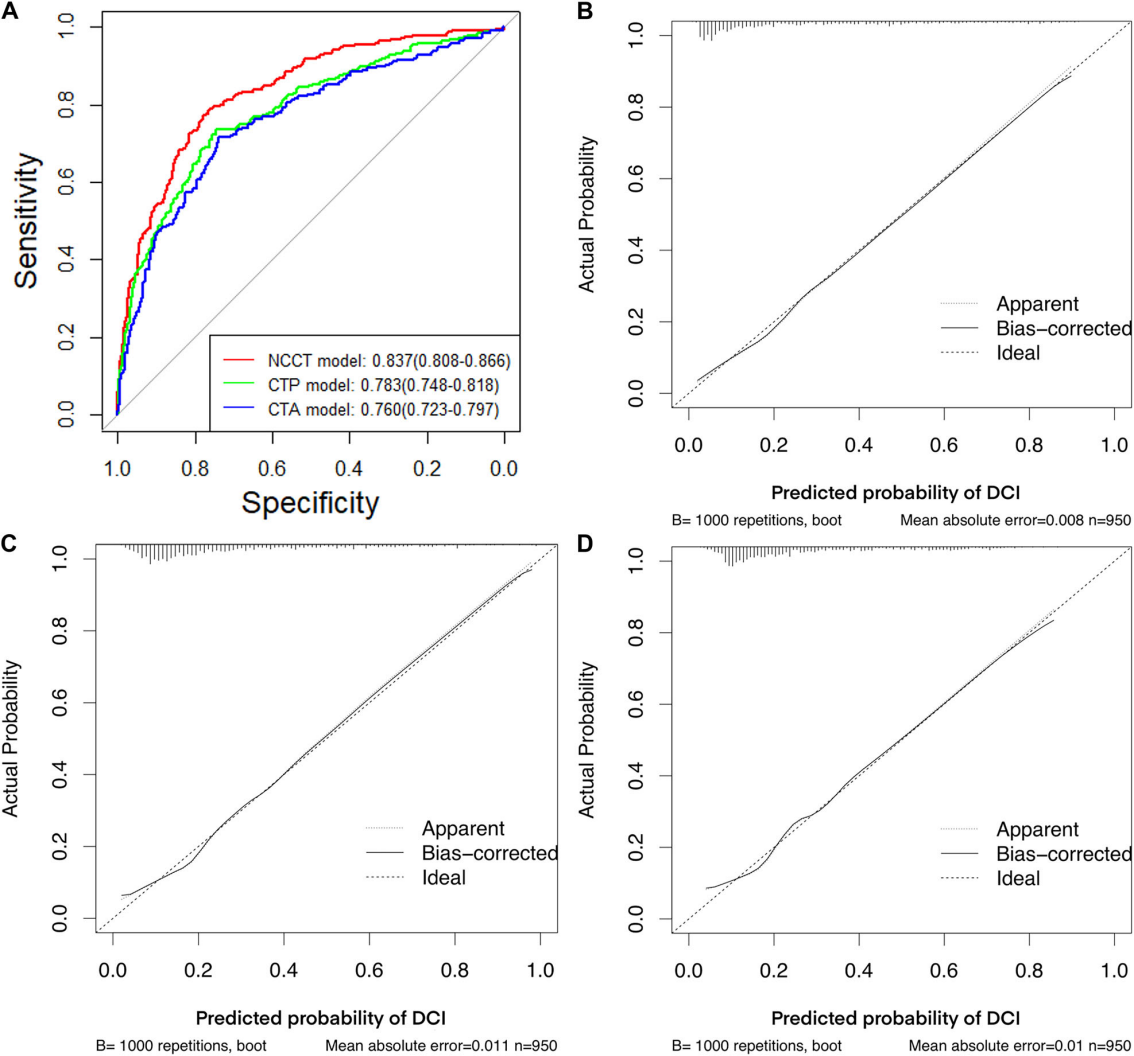
Evaluation of model discrimination and calibration. Receiver operating characteristic curves (**A**) and calibration curves (**B**–**D**) are presented for the non-contrast CT, CT perfusion, and CT angiography models, respectively

Based on the multivariate analysis (Table 2), three logistic regression models were constructed. The discrimination ability was excellent for the NCCT model (AUC: 0.837; 95% CI: 0.808–0.866; *p* < 0.001) and good for the CTP (AUC: 0.783; 95% CI: 0.748–0.818; *p* < 0.001) and CTA (AUC: 0.760; 95% CI: 0.723–0.797; *p* < 0.001) models. The AUC of the NCCT model was significantly higher than that of the CTP (*p* < 0.001) and CTA (*p* < 0.001) models, while the AUC of the CTP model was significantly higher than that of the CTA model (*p* < 0.05). The calibration ability was good for all three models (*p* = 0.835 for the NCCT model, *p* = 0.377 for the CTP model, *p* = 0.096 for the CTA model). The ROC curves and calibration plots for the models are shown in Fig. 3.

**Table 2:** NCCT, CTP, and CTA models

Characteristic	Beta coefficient	OR	95% CI	*p*	Sensitivity	Specificity
NCCT model					0.772	0.778
Age	0.040	1.041	1.023–1.059	< 0.001		
WFNS	0.811	2.249	1.526–3.316	< 0.001		
mFS	0.674	1.963	1.281–3.008	0.002		
IVH	0.521	1.683	1.009–2.807	0.046		
Intracranial hematoma	0.721	2.057	1.307–3.238	0.002		
SEBES	0.853	2.347	1.410–3.905	0.001		
Acute hydrocephalus	1.328	3.775	2.604–5.472	< 0.001		
CTP model					0.736	0.746
Age	0.048	1.049	1.033–1.065	< 0.001		
Sex	−0.443	0.642	0.440–0.936	0.021		
WFNS	1.324	3.760	2.632–5.373	< 0.001		
TMax	0.599	1.820	1.470–2.253	< 0.001		
CTA model					0.715	0.739
Age	0.050	1.051	1.035–1.067	< 0.001		
WFNS	1.688	5.406	3.881–7.530	< 0.001		
Size	0.059	1.061	1.007–1.118	0.027		

*AUC* area under the curve, *CTA* CT angiography, *CTP* CT perfusion, *IVH* intraventricular hemorrhage, *mFS* modified Fisher Score, *NCCT* non-contrast CT, *SEBES* Subarachnoid Hemorrhage Early Brain Edema Score, *TMax* transit time to the center of the impulse response function, *WFNS* World Federation of Neurosurgery Scale

Regarding internal validation, the discrimination ability was excellent for the NCCT model (optimism-adjusted AUC: 0.840; 95% CI: 0.811–0.870) and good for the CTP (optimism-adjusted AUC: 0.785; 95% CI: 0.748–0.820) and CTA (optimism-adjusted AUC: 0.761; 95% CI: 0.724–0.799) models.

### Subgroup analyses

In the good-grade aSAH group, the predictive ability of the NCCT model (AUC: 0.802; 95% CI: 0.757–0.847) was superior to that of the CTP model (AUC: 0.712; 95% CI: 0.659–0.766) (*p* = 0.001). Notably, no significant differences in the CTA variables were observed between the DCI and non-DCI groups among the good-grade aSAH patients. Therefore, a CTA model was not constructed.

In the poor-grade aSAH group, the predictive ability of the NCCT model (AUC: 0.763; 95% CI: 0.704–0.821) was similar to that of the CTP model (AUC: 0.735; 95% CI: 0.674–0.796) (*p* = 0.399). Both of these models had superior predictive ability compared with that of the CTA model (AUC: 0.667; 95% CI: 0.601–0.734) (NCCT vs. CTA: *p* = 0.003; CTP vs. CTA: *p* = 0.009).

## Discussion

Previous studies have demonstrated that several imaging risk factors based on NCCT, CTA, and CTP are predictive of DCI after aSAH [6–13]. However, there is limited knowledge about whether these three imaging modalities have comparable predictive abilities. In this study, NCCT, CTA, and CTP models were constructed for the prediction of DCI, and the predictive abilities of these models were compared. The results showed that the NCCT model, involving easily obtainable variables on admission (age, WFNS, mFS, IVH, intracranial hematoma, SEBES, and acute hydrocephalus), had the best predictive ability for both the entire cohort and the good-grade aSAH group. Meanwhile, there was no difference in predictive ability between the NCCT and CTP models for the poor-grade aSAH group.

NCCT is more cost-effective and readily available than advanced imaging modalities, and provides multiple radiological scales associated with DCI. Two predictive models have been developed by combining clinical and NCCT variables, namely the EDCI score (WFNS, mFS, SEBES, and IVH) [21] and the Practical Risk Chart (WFNS, mFS, Hijdra, and age) [22], with both models demonstrating good discrimination ability. The NCCT model created in this study supports the previous two models, thus increasing confidence in the use of NCCT in clinical practice. More importantly, the prediction ability of the NCCT model (AUC: 0.802; 95% CI: 0.757–0.847) was significantly improved compared with that of the EDCI score (AUC: 0.785; 95% CI: 0.752–0.815) and the Practical Risk Chart (AUC: 0.63; 95% CI: 0.57–0.69). These findings confirm that the NCCT model is accurate and useful for identifying aSAH patients at high risk for developing DCI based on clinical and radiographic data on admission. Although some previous studies compared the predictive values of individual NCCT-based radiological scales [23, 24], a single radiological scale is inevitably one-sided, whereas the NCCT model integrates clinical and imaging data to circumvent this limitation. Thus, the NCCT model can enable better risk stratification and management.

CTP is useful for assessing the early cerebral hemodynamic status after aneurysm rupture [13]. Although some small, single-center studies have proposed optimal cutoff values for different perfusion parameters to predict DCI [17, 25–28], the results were heterogeneous and have not been validated in large external cohorts. Meanwhile, the lack of standardized scanning and post-processing protocols may lead to significant differences in CTP results [29]. It is also worth noting that CTP results are vulnerable to motion artifacts [14], especially in aSAH patients, who are often restless and find it difficult to cooperate with long scanning procedures. Despite this, our subgroup analyses showed that the predictive ability of the CTP model was similar to that of the NCCT model in the high-grade aSAH group. A possible explanation for this finding may be the complexity of the pathophysiology in DCI, because the microcirculatory dysfunction may be more pronounced in poor-grade aSAH patients, and the NCCT model cannot adequately reflect these changes [13, 16]. Another possible explanation is that poor-grade aSAH patients with a decreased level of consciousness may have more accurate CTP results due to the absence of significant motion artifacts.

CTA provides the characteristics of a ruptured aneurysm and can detect CVS. However, it is difficult to detect a distal vasospasm caused by a microthrombus [13, 30]. We found that CVS on admission had no significant correlation with DCI in either the univariate or multivariate analyses. These findings confirm previous data showing that DCI often occurs in patients without CVS [30]. Therefore, our finding that the predictive ability of the CTA model was significantly lower than that of the NCCT and CTP models was not unexpected. The present findings also highlight the predictive value of CTP for the detection of ischemic tissues remote from the site of CVS [13].

This study has some limitations. First, it was a single-center retrospective observational study, and although it had a relatively large sample size, potential selection bias was inevitable. Second, the lack of external validation may limit the applicability of the findings. Third, considering the clinical applicability, we included only the most representative mFS in the NCCT model, rather than several hemorrhage volume scores, such as the BNI, Hijdra, and Claassen scales.

## Conclusions

Overall, the NCCT model showed excellent performance in predicting DCI at admission and had better predictive ability than the CTP and CTA models. Notably, the predictive ability of the NCCT and CTP models was comparable in patients with poor-grade aSAH. Our results suggest that NCCT may serve as a surrogate marker for DCI when advanced imaging is unavailable or not feasible. Validation in prospective studies is needed to confirm the clinical utility of our findings in practice.

## Data Availability

Anonymized data from the study are available from the corresponding author upon reasonable request.

## Abbreviations

aSAH: Aneurysmal subarachnoid hemorrhage
AUC: Area under the curve
CBF: Cerebral blood flow
CBV: Cerebral blood volume
CTA: CT angiography
CTP: CT perfusion
CVS: Cerebral vasospasm
DCI: Delayed cerebral ischemia
H-H: Hunt-Hess grade
IVH: Intraventricular hemorrhage
mFS: modified Fisher Score
MTT: Mean transit time
NCCT: Non-contrast CT
ROC: Receiver operating characteristic
SEBES: Subarachnoid Hemorrhage Early Brain Edema Score
TMax: Transit time to the center of the impulse response function
TTD: Time to delay
TTS: Time to start
WFNS: World Federation of Neurosurgery Scale
